# Intraosseous hemangioma with aneurysmal bone cyst-like changes of the hyoid bone: Case report and literature review

**DOI:** 10.1097/MD.0000000000037137

**Published:** 2024-02-09

**Authors:** Jeonghyun Oh, Song Iy Han, Sung-Chul Lim

**Affiliations:** a Department of Otorhinolaryngology, Chosun University, Gwangju, Korea; b Division of Premedical Science, Chosun University, Gwangju, Korea; c Department of Pathology, College of Medicine, Chosun University, Gwangju, Korea.

**Keywords:** ABC-like changes, hyoid, intraosseous hemangioma

## Abstract

**Rationale:**

Intraosseous hemangioma is a rare benign vascular tumor of the bone that can affect any body part; however, the most common site is the vertebra, followed by calvarial bones.

**Patient concerns:**

We present a case of intraosseous hemangioma in a 23-year-old male who presented a feeling of fullness in the throat for 3 months. The hyoid bone level had a hard mass of about 5 cm. Fine needle aspiration showed 5 mL dark bloody aspirates. Magnetic resonance image showed a 5.3 cm mixed signal intensity lesion in the hyoid body.

**Diagnosis:**

Histopathologic examination showed intraosseous hemangioma with aneurysmal bone cyst (ABC)-like changes in the hyoid bone.

**Interventions:**

The mass was completely removed without significant problems.

**Outcomes:**

Complete mass excision and symptomatic improvements were achieved, and no subsequent relapses were observed.

**Lessons:**

The authors experienced a case of intraosseous hemangioma with ABC-like changes. There has been no case report of intraosseous hemangioma in the hyoid bone. This case showed a spectral pattern of the ABC-like changes developing from the underlying bone tumor as a secondary change. ABC-like changes in bone tumors can mislead the diagnosis. Careful examination of the tumor is essential for the correct diagnosis of ABC or ABC-like changes.

## 1. Introduction

Intraosseous hemangioma (IOH) is a rare tumor, accounting for <1% of bone tumor cases. IOH grows slowly and more commonly occurs in women; furthermore, it is typically found in vertebrae and skull bones. Additionally, it occurs in long bones, tubular bones, and ribs.^[1]^ Most IOH cases show characteristic radiographic findings due to the formation of reactive spicules caused by the lesion.^[2]^ Clinical symptoms of IOH are wide-ranging depending on lesion size and location, from asymptomatic cases in which IOH is found by chance to symptomatic ones in which pathological fractures are visible.^[3]^ Since the lesion can manifest diverse morphologies, diagnosing IOH is difficult. To accurately diagnose IOH, it is essential to perform a histologic examination. With image studies only, differential diagnosis of other benign and malignant tumors (such as fibrous dysplasia, eosinophilic granuloma, aneurysmal bone cyst [ABC], Ewing sarcoma, chondrosarcoma, and metastasis) should be considered.^[4]^

ABC is a benign blood-filled cystic neoplasm in bones. The condition is rare, accounting for 2.5% of all bone tumors.^[5]^ The occurrence of ABC does not differ by sex, and in most cases, it occurs in the first 2 decades of life, when the skeletal system is not yet fully developed.^[6]^ Previously, ABC was believed to occur as a reaction to traumas or a reactive lesion due to underlying vascular events. In current opinion, however, it is regarded as a true neoplasm due to the rearrangement of the *ubiquitin-specific peptidase 6 (USP6*) gene.^[5,7]^

ABC-like changes, previously called secondary ABC, are areas showing hemorrhagic cystic change in benign or malignant bone tumors. The changes occur due to the disruption of osseous circulation caused by comorbidity and are typically found in giant cell tumors, chondroblastoma, osteoblastoma, osteosarcoma, chondromyxoid fibroma, and fibrous dysplasia.^[5,8]^ Therefore, in cases where ABC-like changes are dominant, thorough sampling and careful examination should be performed, considering that an underlying bone tumor is masquerading.

This case study presents the first rare case of IOH in the hyoid bone. In addition, considering that clinical and pathological diagnoses may not be straightforward if IOH accompanies ABC-like changes, the authors report a literature review of relevant findings.

## 2. Case report

**Figure 1.**
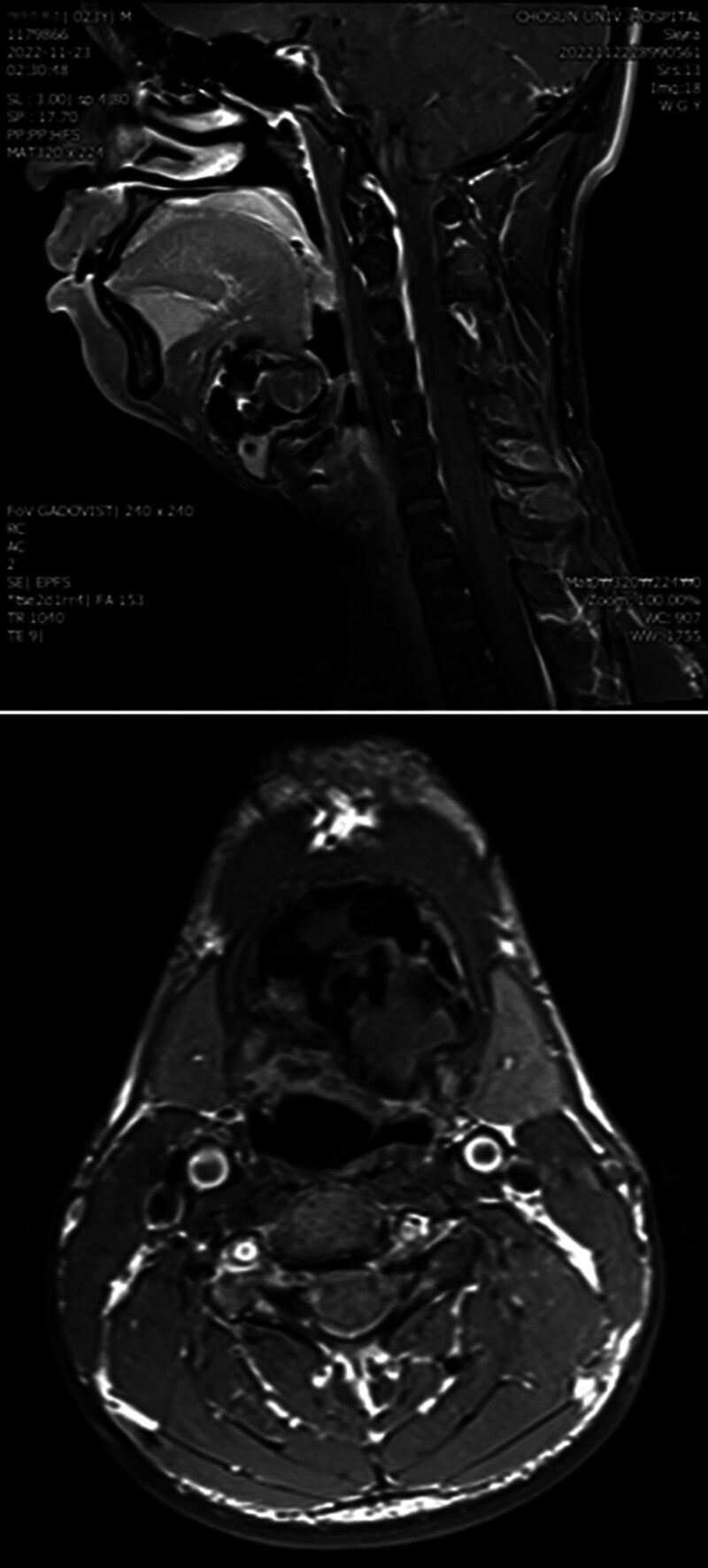
MRI revealing a 5.3 × 4.3 cm mass located in the upper anterior neck. The lesion exhibited mixed signal intensity, consisting of solid components and cystic areas, as depicted in the sagittal view (upper) and axial view (lower) images. MRI = magnetic resonance image.

A 23-year-old man visited the otorhinolaryngology department complaining of feeling fullness in the throat. The feeling began 3 months before. In palpation of the neck, a hard mass sized approximately 5 cm was felt at the hyoid bone level. The patient did not present any notable medical or family histories. In fine needle aspiration, a needle was inserted into hard tissue and was used to collect approximately 5 mL of dark bloody aspirates. Thyroglossal duct cyst or malignancy was suspected, so excision was planned, and neck magnetic resonance imaging (MRI) was performed. T1/T2 weighted imaging tests showed a mixed signal intensity lesion of 5.3 cm in maximal diameter in the upper anterior neck. Both solid lesions and cystic components were present in the lesion, and calcification was observed. Partially restricted diffusion was also found. No detectable body of hyoid bone was found, so the lesion seemed to have originated in the body of the hyoid bone. Cervical lymphadenopathy was not observed (Fig. 1).

Based on the MRI findings, it was determined that a differential diagnosis should be made with bone tumors in the hyoid bone, such as enchondroma, low-grade sarcoma, and complicated thyroglossal duct cyst.

The patient underwent preoperative check to perform *en bloc* resection under general anesthesia. Other than aspartate transaminase measuring 46 IU/L and alanine transaminase measuring 104 IU/L were no abnormal findings. After liver functions improved, surgery was performed.

To remove the lesion formed around the hyoid bone, an attempt was made to separate the periosteum from the surrounding soft tissue. However, separation was infeasible due to a serious periosteal reaction and heavy bleeding. Hence, the lesion and soft tissue around the hyoid bone and some muscular tissues were resected.

The specimen obtained from the resection was sized 5.9 × 4.5 × 3.2 cm, and on the surface, the size of the lesion appeared to be 4.3 × 3.2 cm. Furthermore, the margin’s boundary was irregular, the cut surface was black and yellow discolored, and several cystic structures of various sizes were observed.

The histopathologic exam showed growth of small-to-medium-sized dilated thin-walled blood vessels in intratrabecular space. Most vessels were filled with red blood cells (RBC), and the vascular wall was thin as if lined with endothelial cells only.

The exam showed a mixture of sites at which primarily vascular proliferation was observed. At the same time, connective tissues were hardly seen, sites at which the volume of connective tissues gradually increased while vascular density reduced, and sites at which little vascular proliferation was found while fibroblasts markedly increased. At many sites in the lesion, osteoclast-type giant cells were found. The cells were seen sporadically at sites where primary vascular proliferation was observed but were very common at sites where fibroblasts markedly increased. Hemosiderin pigmentation was also commonly seen at the sites with substantially increased fibroblasts.

**Figure 2.**
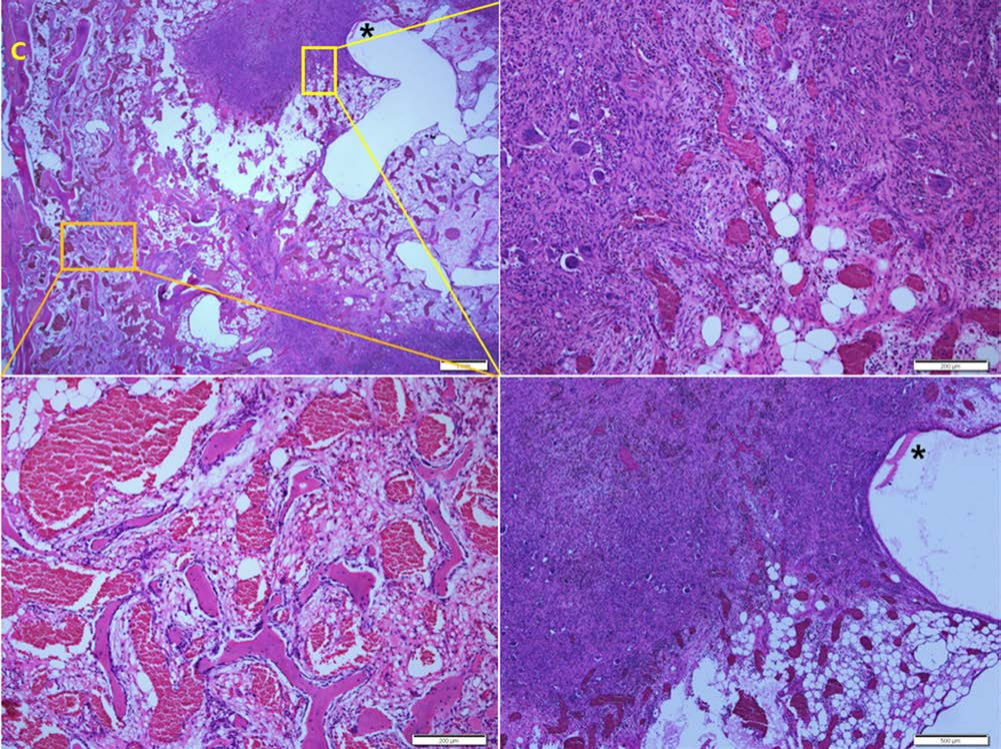
Histopathological examination of the lesion. The scanning view (upper left) displayed proliferation of small-sized dilated thin-walled blood vessels within the intratrabecular and endosteal spaces. The lesion exhibited dense fibroblastic proliferative foci and cystic spaces of varying sizes. The cortical bone (C) exhibited irregular thinning and disruption caused by the vascular proliferative lesion. Scale bar measures 1 mm. At higher magnification of the orange boxed area (lower left), dilated thin-walled blood vessels filled with red cells were observed. Scale bar measures 200 µm. Further magnification of the yellow boxed area (upper right) revealed proliferative blood vessels interspersed with fibroblastic spindle cell proliferation and scattered osteoclastic giant cells. Scale bar measures 200 µm. Additionally, at higher magnification of the asterisk-marked area in the scanning view (lower right), a dilated cystic space and adjacent fibroblastic proliferative lesion containing scattered osteoclastic giant cells, hemosiderin pigments, and dilated blood vessels were observed. Scale bar measures 500 µm.

The lesion caused thinning of the cortical bone in an extensive area, penetrated the bone, and was directly connected to surrounding soft tissue at several sites. In the lamellar trabecular and cortical bones in contact with the lesion, newly formed woven bones were abundant. It was determined that they reflected reactive change because neither cytologic atypia nor mitosis was found. Neither cytologic atypia nor mitosis were found in vascular endothelial cells and intervascular tissues, too (Fig. 2).

**Figure 3.**
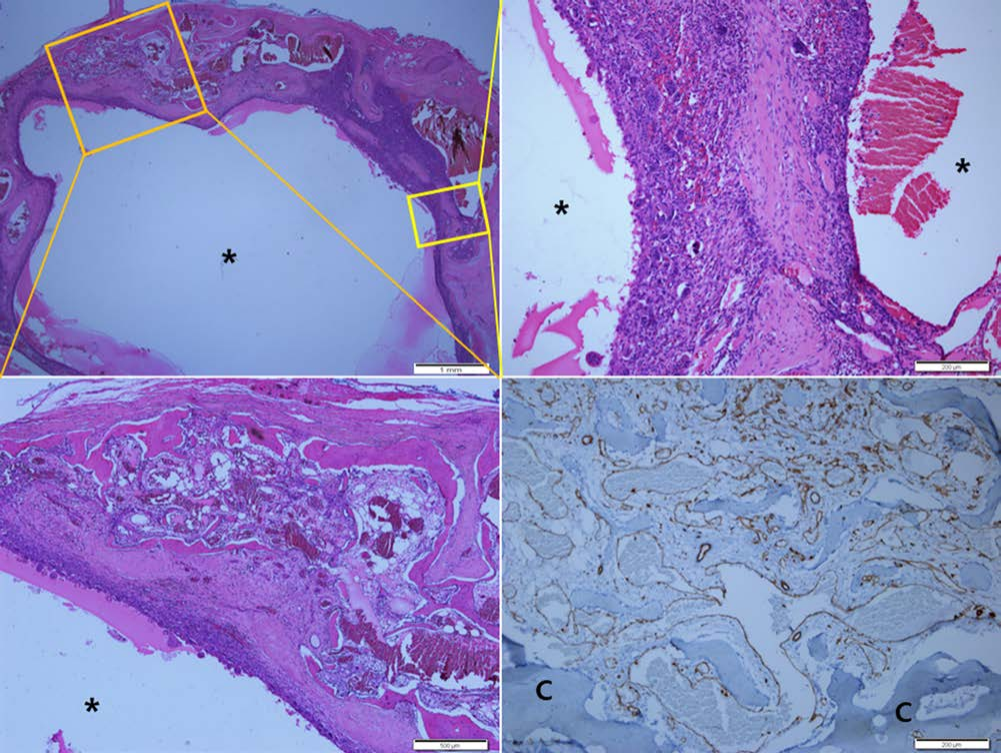
Histopathological examination of the cystic lesions (asterisks). The scanning view (upper left) displayed a macrocystic space filled with pinkish proteinaceous fluid, lacking distinctive endothelial linings. Proliferation of variable-sized dilated thin-walled blood vessels was identified surrounding the cyst. Scale bar measures 1 mm. At higher magnification of the orange boxed area (lower left), dilated thin-walled blood vessels filled with red cells were observed, accompanied by adjacent cortical destruction. Scale bar measures 500 µm. Further magnification of the yellow boxed area (upper right) revealed cystic spaces (asterisks) containing pinkish proteinaceous material and red cells. The cysts were lined by fibroblastic spindle cell proliferation with scattered osteoclastic giant cells. Scale bar measures 200 µm. Immunohistochemical staining (lower right) demonstrated CD31 positivity in the thin-walled vascular proliferation within the intratrabecular space. Cortical thinning (C) was observed. Scale bar measures 200 µm.

Of the several cysts found in the lesion, most contained pinkish proteinaceous fluid, and RBC was present in some cysts. There were small fragments of cellular septa containing fibroblast-like spindle cells, many osteoclast-like giant cells, and reactive woven bone with osteoblastic rimming. Although cell lining was not found in most cystic spaces, areas that appeared flattened endothelial-like cells were visible in some cystic spaces. However, they tested negative for CD31, CD34. At sites where the growth of small-to-medium-sized dilated thin-walled blood vessels was found, the cell lining in intravascular walls was positive for CD31 and CD34 and negative for D2-40 on immunohistochemical staining (Fig. 3). The findings above were determined to be consistent with IOH with ABC-like changes.

**Figure 4.**
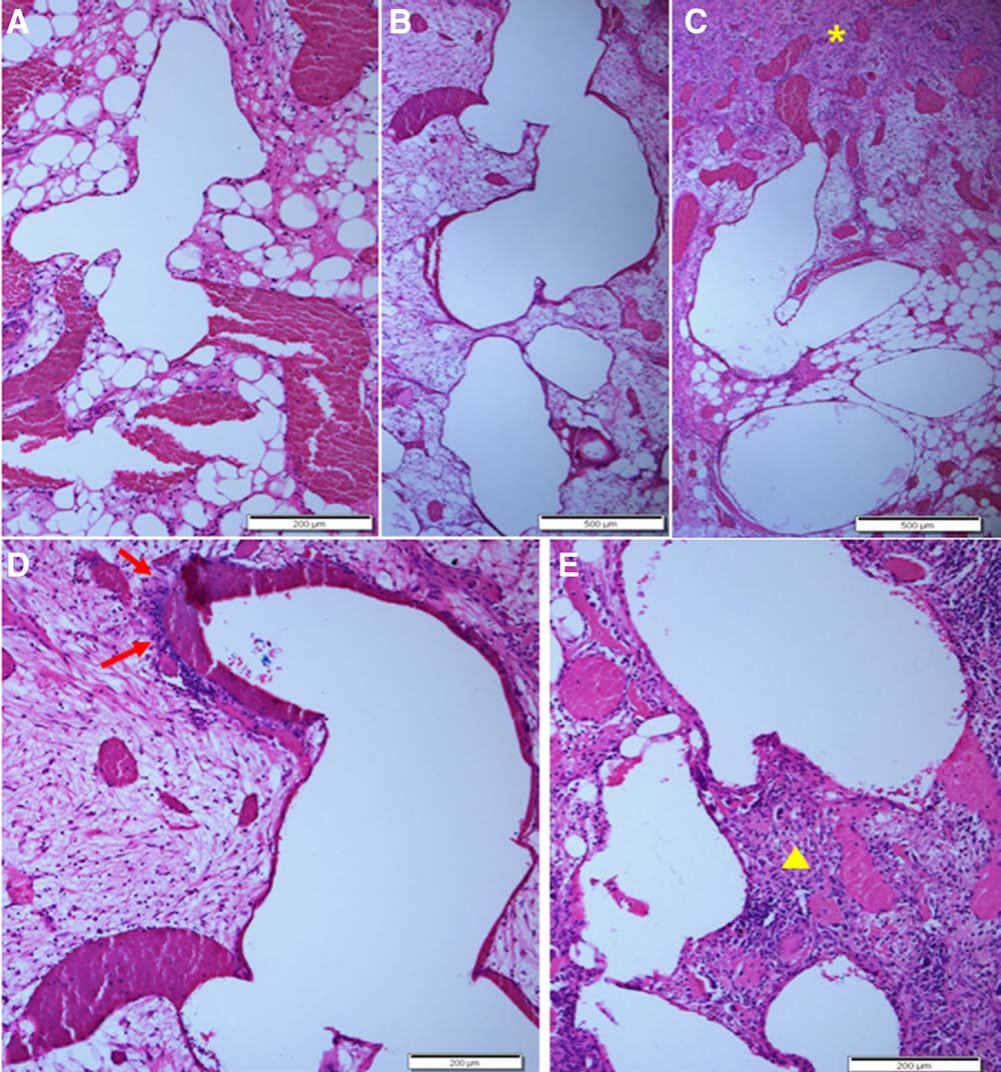
Spectral findings of cystic development. (A) Tortuous spaces partially filled with red cells were identified. (B) Larger cystic spaces were observed. (C) Larger cystic spaces and adjacent fibrotic area (asterisk) with some inflammatory cells were present. (D) Cystic dilated space containing red cells and pericystic inflammation (arrows) were noted in the loose fibrous area. (E) Variable-shaped and sized cystic spaces, along with adjacent dilated vessels filled with red cells, were identified. Pericystic chronic inflammation with fibrosis (pyramid) was observed. Scale bars measure 200 µm (A, D, E) and 500 µm (B, C).

In a close inspection of the sites primarily showing vascular proliferation, small and large cysts (microcysts) were sporadically observed, and the size of cysts tended to increase (macrocysts) as the volume of connective tissues increased. Based on cysts of varying shapes and sizes found at many sites in the lesion, it was suggested that spectral cystic change occurred due to some events in the course of IOH development. In other words, the authors speculated the plausibility that ABC-like changes were induced throughout the process, beginning with the initial microcystic change through inflammation to reactive fibrosis (Fig. 4).

Of the paraffin blocks not subjected to the decalcification process, those containing a cystic lesion were selected to perform the *USP6* gene rearrangement test by using fluorescence in situ hybridization. *USP6* gene rearrangement was not found; thus, the cystic changes were determined to be ABC-like changes rather than ABC.

No signs of recurrence have been observed after 12 months of surgery with computed tomography (CT) follow-up.

Based on all the findings, the case was finally diagnosed as IOH with ABC-like changes in the hyoid bone.

## 3. Discussion

Whereas hemangioma rarely occurs in bones, 35% of vascular malformations are found in bones. Therefore, if a vascular lesion is found in bones, caution should be taken in diagnosing it as IOH.^[9]^ IOH mainly occurs in vertebrae and cranial bones, and cases of IOH in the hyoid bone have never been reported. The peak incidence of IOH is found in the fourth and fifth decades of life, and the condition is more prevalent in women than in men by approximately 3 times.^[10,11]^

In most IOH cases, the pathogenesis is unknown. Local trauma and menopause are discussed as possible causes, but a causal relationship has not been conclusively established. The present case had no trauma or history of surgery in the affected area.^[12,13]^

IOH grows slowly, so it is clinically silent and discovered only when it grows large enough to show symptoms and cause dysfunctions. IOH tends to be asymptomatic in cases in which it occurs in the axial skeleton and symptomatic in cases in which it occurs in the appendicular skeleton. The present case did not complain of pain but felt fullness in the throat.^[14]^

CT is the imaging modality of choice in diagnosing IOH because it can delineate the margins of the lesion, illuminate the characteristic “soap bubble” or “honeycombed” appearance, and clearly show the “sunburst” pattern of radiating trabeculations.^[15]^

In atypical IOH, it is more difficult to make a radiographic diagnosis because the likelihood of fibrous dysplasia, ABC, eosinophilic granuloma, multiple myeloma, chondrosarcoma, Ewing sarcoma, metastatic tumor, and other benign and malignant lesions should also be considered.^[16]^

Fine needle aspiration is of little help in diagnosing IOH. The reason is that it is not easy to perform aspiration in a bony lesion, the procedure may cause postprocedure bleeding, and it is highly likely for the specimens to contain nondiagnostic normal RBC alone.^[17]^ In the present case, too, only normal RBC was aspirated.

Preoperative embolization and sclerotherapy are performed to reduce the risk of intraoperative bleeding. However, neither is needed if complete surgical resection is planned.^[18]^

Although how to treat IOH (ranging from biopsy to segmental resection) is controversial, surgical treatment is not necessary for the treatment of small asymptomatic IOH in long bones. Curettage and bone grafting are typically performed in cases with a large symptomatic lesion or a pathologic fracture. However, some studies reported that radical *en bloc* resection was necessary to prevent recurrence and massive bleeding.^[19,20]^ In the present case, it was decided to perform *en bloc* resection based on lesion location, tumor extent, and structural characteristics of the hyoid bone. Moreover, the outcome was favorable.

ABC, a benign blood-filled cystic neoplasm of bone, is a rare type of tumor, accounting for 2.5% of all bone tumor cases.^[21]^ The incidence of ABC does not differ between sexes.^[22]^ The condition most commonly occurs in patients in whom the skeletal system is not fully developed, with the highest incidence in the first 2 decades of life.^[6]^ In contrast, ABC-like changes indicate a reactive process. These changes are often observed as secondary findings in cases involving underlying vascular events, increased venous blood flow, or as a response to previous trauma.^[23]^

In the present case, it was confirmed that from typical IOH, connective tissues increased, that the closer to the sites where aggressive growth of fibroblasts without vascular proliferation was primarily observed, the larger the cysts without lining cells (microcysts), and that eventually, macrocysts were produced, which were thought to be ABC-like changes. Based on the spectral change, it was inferred that ABC-like changes reflected a reactive process emerging in the occurrence of some events to the underlying disease (i.e. minor trauma and physical stresses).

ABC radiographic features are very clear and diagnostic. In conventional radiographs, a finding of an eccentric radiolucent lesion with the expansile remodeling of bone can be observed, a thin surrounding rim of the periosteum and subperiosteal bone be found, and a multilocular appearance be seen. On CT, ABC appears to be a well-delineated lytic lesion; in most cases, a thin surrounding rim consisting of reactive bones is present. Sometimes, fluid-fluid levels may be seen, and MRI should be performed to most clearly view them. Generally, a cystic lesion shows variable signal intensity with a rim of low T1 and T2 signal.^[24,25]^

No characteristic histopathological findings or immunohistochemical markers distinguish between ABC and ABC-like changes, making it difficult to differentiate one from the other. However, a *USP6* gene rearrangement study is of help in differentiating them because ABC is a true neoplasm-inducing *USP6* gene rearrangement.^[5,7]^

Regarding the present case, it is speculated that ABC-like changes were caused by unidentified events (including minor trauma) during IOH development due to repeated hemorrhagic cystic change since the changes were found in the presence of IOH and *USP6* gene analysis of the cystic lesion did not show gene rearrangement.

ABC-like changes can masquerade an underlying tumor. Therefore, it is essential to perform thorough sampling and careful examination to determine the presence or absence of various benign and malignant underlying bone tumors that may induce ABC-like changes.

## 4. Conclusion

The current study is the first case report of IOH in the hyoid bone. In addition, the present case study adds to the rarity, as ABC-like changes accompanied IOH. The case showed a spectral pattern of ABC-like changes suggesting a secondary change induced by an underlying bone tumor.

## Author contributions

**Data curation:** Jeonghyun Oh, Song Iy Han.

**Funding acquisition:** Sung-Chul Lim.

**Investigation:** Jeonghyun Oh.

**Methodology:** Jeonghyun Oh, Song Iy Han.

**Supervision:** Sung-Chul Lim.

**Validation:** Sung-Chul Lim.

**Writing – original draft:** Jeonghyun Oh.

**Writing – review & editing:** Song Iy Han, Sung-Chul Lim.
